# Supplementary figures and images for: Proximity interactome of alphavirus replicase component nsP3 includes proviral host factors eIF4G and AHNAK
Source: PLoS Pathog. 2025 Apr 7;21(4):e1013050. doi: 10.1371/journal.ppat.1013050 (PMC12005498; doi:10.1371/journal.ppat.1013050)

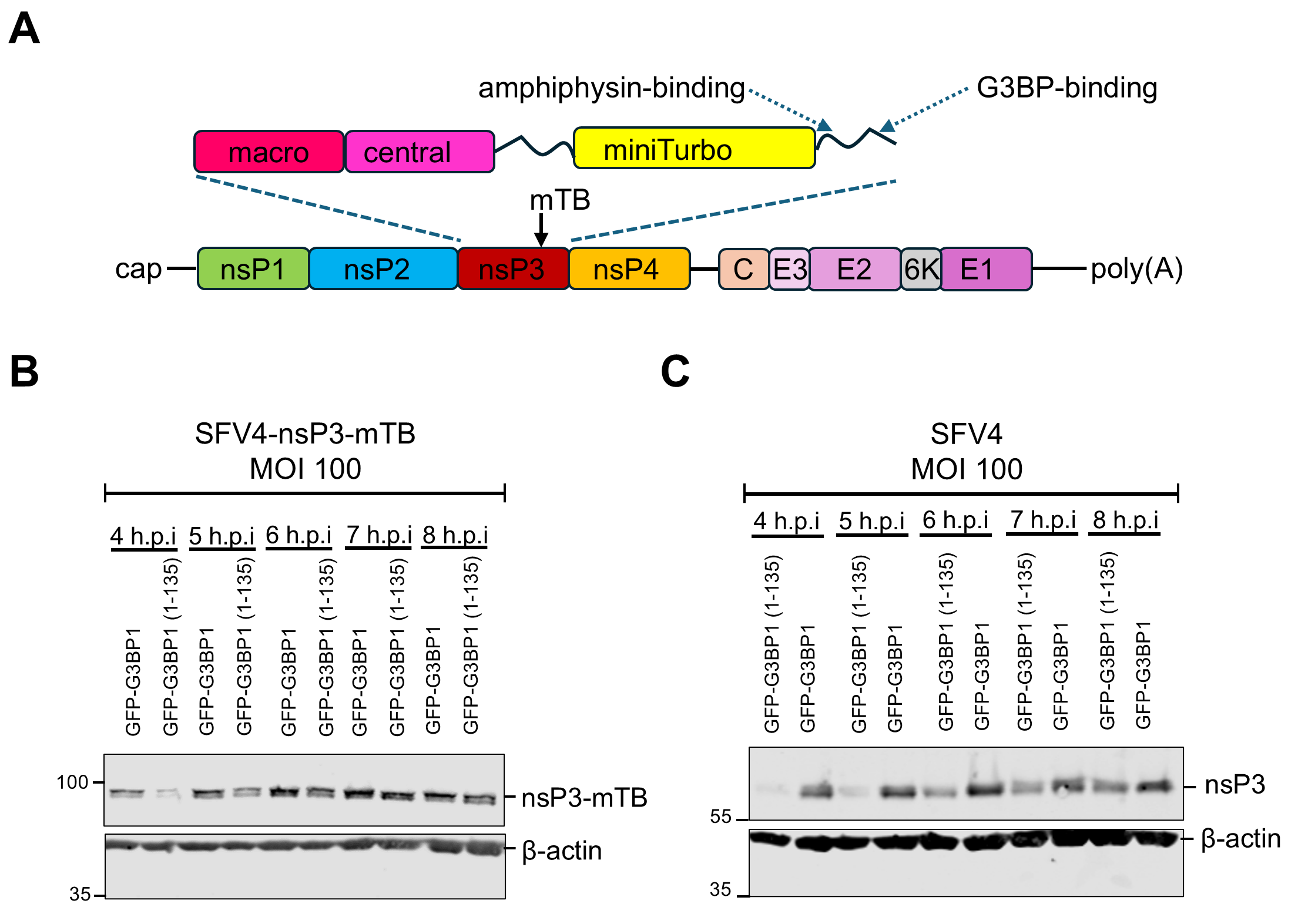

Supplement: S1 Fig — A) mTB was inserted to the SFV4 genome (bottom), so that it is located approximately at the center of the C-terminal unstructured region of nsP3 (shown as squiggly lines), followed by the known binding sites for amphiphysin (BIN1) and G3BPs (top). B and C) GFP-G3BP1 and GFP-G3BP1(1-135) cells were infected with either (B) SFV4-nsP3-mTB or (C) SFV4 at MOI 100. Cell lysates were collected at the indicated time points, and analyzed for nsP3/nsP3-mTB expression by Western blotting using antibodies against nsP3; β-actin was used as a loading control. (TIF) [file ppat.1013050.s001.tif]

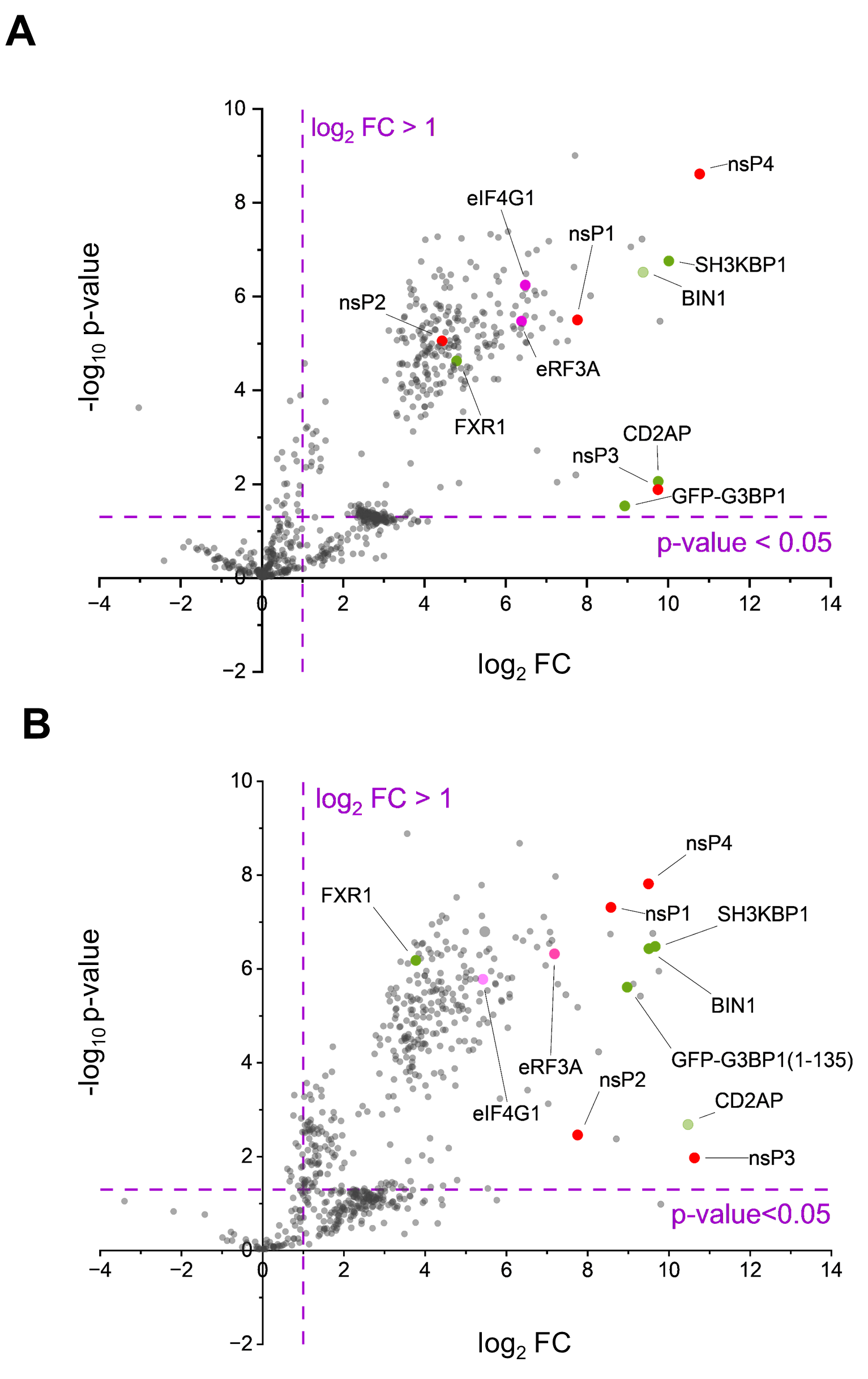

Supplement: S2 Fig — A and B) Volcano plots represent the distribution of the proteins detected in GFP-G3BP1 cells (A) and GFP-G3BP1(1-135) cells (B) through proteomic analysis. High confidence interactors were determined as log2 Fold change > 1 and -log10 adjusted p-value >1.3 (i.e. p-value < 0.05; independent Student’s t-test), indicated with the shaded area. The viral non-structural proteins in the dataset are marked with red, the most prominent previously known interactors are marked with green, and the two translation factors are indicated in yellow. (TIF) [file ppat.1013050.s002.tif]

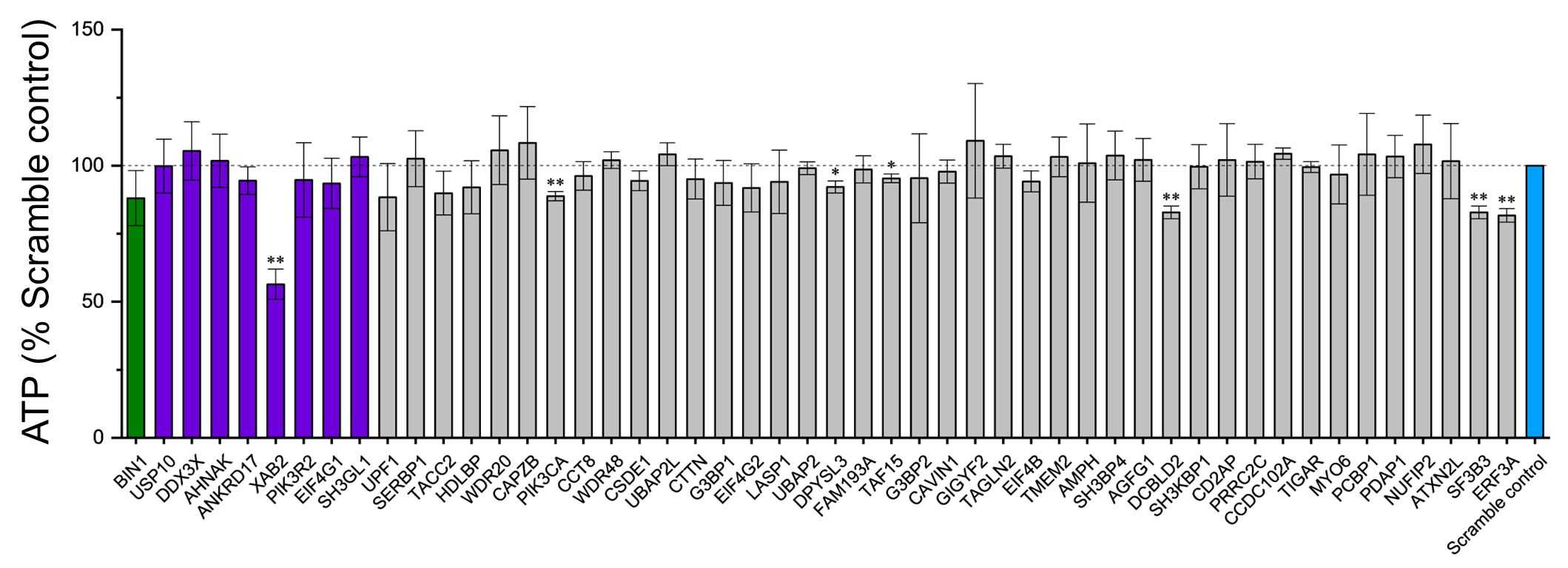

Supplement: S3 Fig — The luminescence levels were determined using CellTiter Glo 2.0 (measuring cellular ATP levels) in U2OS cells, and normalized to those treated with non-targeting scramble control siRNA. The graph is an average of three independent biological replicates and the error bars indicating the standard deviation. The p-values were calculated using independent Student’s t-test; * p<0.05 and ** p<0.01. (TIF) [file ppat.1013050.s003.tif]

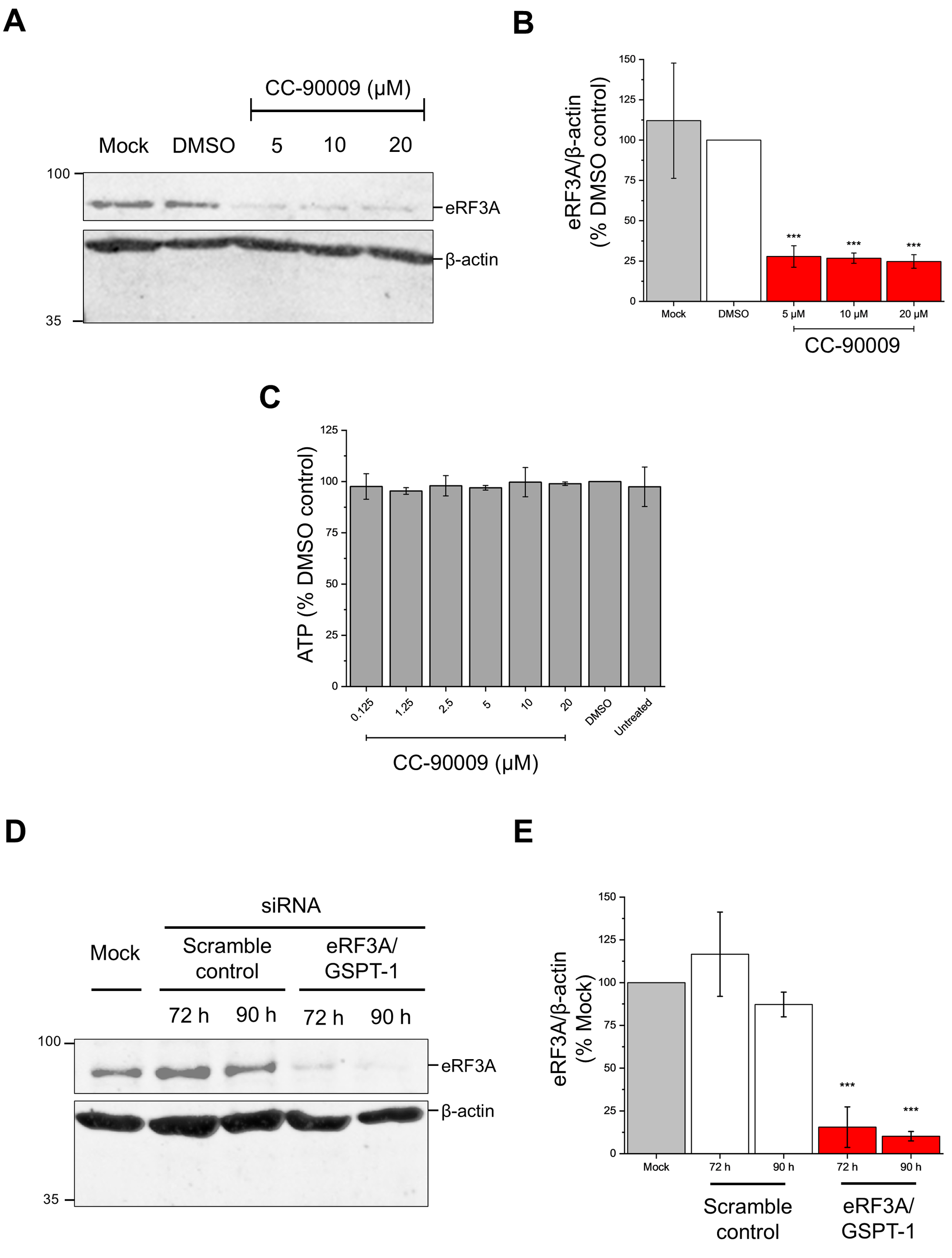

Supplement: S4 Fig — A) U2OS cells were treated with the indicated concentrations of CC-90009 (see Materials and Methods), and cell lysates were analyzed by Western blotting for eRF3A. B) Quantitation of eRF3A expression based on three independent experiments. C) Cell viability of U2OS cells after CC-90009 treatment. The luminescence levels were determined using CellTiter Glo 2.0 (measuring cellular ATP levels). D) U2OS cells were treated with siRNA against eRF3A or with the scramble control, and cell lysates were analyzed by Western blotting for eRF3A. The time points 72 h and 90 h represent the start and end points of the corresponding SFV infection experiments. E) Quantification of eRF3A expression based on three independent experiments. The error bars indicate standard deviation. The p-values were calculated using independent Student’s t-test; *** p<0.001. (TIF) [file ppat.1013050.s004.tif]
