# Supplementary material for: Proximity interactome of alphavirus replicase component nsP3 includes proviral host factors eIF4G and AHNAK
Source: PLoS Pathog. 2025 Apr 7;21(4):e1013050. doi: 10.1371/journal.ppat.1013050 (PMC12005498; doi:10.1371/journal.ppat.1013050)
Supplement: Attachment — Submitted filename: Rebuttal letter Thiruvaiyaru et al.docx [file ppat.1013050.s012.docx]

**Thiruvaiyaru et al., responses to Reviewers**

**Additional Editor Comments:**

All reviewers were enthusiastic about your work and made helpful suggestions to improve the manuscript. I share their view that the work has an innovative experimental approach and advances the field of alphavirology with regard to the elusive functions of the nsP3 protein.

Rev#2 has made import suggestions to quantify expression levels of antiviral proteins and restructure several Figures with the aim to better support the conclusions. Some additional experimentation may be needed to address these concerns.

Response: We thank the three Reviewers for their helpful comments and for careful reading of the manuscript. All the comments have helped us to make the manuscript easier to follow for the eventual readers. Especially the comments that improve the presentation of the results in Figures are much appreciated. Specific responses, including the major revisions in response to Rev#2, can be found below. The line numbers refer to the marked-up manuscript, where the revisions are highlighted.

Addition: We have added a few sentences (lines 201-204 and 244.245) concerning the recently published results of Kamel et al. (new reference 44). They find that nuclear RNA-binding proteins bind to Sindbis virus RNA in the cytoplasm during virus infection. Prominent among these is the SF3B complex, which includes the SF3B3 protein that was one of the few antiviral proteins we discovered in our SFV data. Kamel et al show that the SF3B complex is antiviral against Sindbis virus and SFV but that it has no effect against CHIKV (their supplementary figure S7, which is not quantitated, could even suggest a proviral effect for CHIKV). Our siRNA data indicates that SF3B3 is proviral for CHIKV. Both Kamel et al and we show that SF3B3 has differential roles for different alphaviruses.

**Reviewers' Comments:**

Reviewer's Responses to Questions

**Part I - Summary**
Please use this section to discuss strengths/weaknesses of study, novelty/significance, general execution and scholarship.

Reviewer #1: In the manuscript: “Proximity interactome of alphavirus replicase component nsP3 includes proviral host factors eIF4G and AHNAK” the authors describe creating SFV which contains an in frame insertion of biotin ligase miniTurbo within non-structural protein 3. Subsequent infections resulted in the biotinylation of viral and host proteins in the proximity of nsP3. With this strategy the authors identified many host proteins that reside in the proximity of nsP3 during the early phase of SFV replication. Subsequently, with an siRNA approach the role of top interactors in SFV and CHIKV replication was identified. The role of nsP3 in alphavirus replication remains enigmatic, therefore this explorative study and the identified putative virus-host interactions can be a starting point for novel studies focussing on alphavirus replication or the identification of antivirals. I do have a few suggestions that may improve the manuscript

Response: The specific responses can be found in the next section. The line numbers refer to the marked-up manuscript.

Reviewer #2: In this manuscript, Thiruvaiyaru et al. have identified novel interactors of alphavirus protein nsP3 by applying a proximity labelling methodology within an authentic virus context. Replication of viral RNA is achieved by a complex interplay of the viral non-structural proteins nsP1, nsP2, nsP3, and nsP4 and host factors and takes place within membranous invaginations into the plasma membrane, termed spherules. To build upon the library of known alphavirus replication-associated factors, the authors determined the interactome of Semliki Forest virus (SFV) nsP3 fused to miniTurbo in baby hamster kidney (BHK) and human osteosarcoma cells (U2OS). The top hits were included in a knockdown siRNA screen in U2OS cells, which revealed eight proviral interactors whose knockdown decreased SFV infection by 50% and five antiviral interactors whose knockdown increased SFV infection. The effect on SFV infection of the proviral translation initiation factor eIF4G1 and antiviral translation termination factor eRF3A were assessed by pharmacological inhibition, revealing a strong proviral effect of eIF4G1 whereas manipulation of eRF3A levels had no effect. The authors are the first to report proximity biotinylation within an authentic alphavirus context to determine nsP3 interactors, which expands upon the current knowledge of SFV replication and translation. The manuscript is written clearly and offers a strong theoretical framework. However, the second half of the manuscript contains experiments in which important controls are absent which precludes drawing the authors’ conclusions regarding translation initiation. Furthermore, the choice of placing certain data in the main manuscript and other important data in the supporting information undermines the coherence of the result section. Hence, the manuscript should be reinforced with control experiments and the data should be restructured as outlined below, before it is suitable for publication.

Response: We agree with the reviewer on the inclusion of controls and the reformatting of the main and supplementary figures. The specific responses can be found in the next section. The line numbers refer to the marked-up manuscript.

Reviewer #3: Thiruvaiyaru and colleagues describe a proteomics screen for interactors of the alphaviral eiigmantic nsP3 protein using a recombinant biotin-transferase tagged Semliki Forest Virus (SFV) and proximity biotinylation/MS approach. The study is flawless, well-conceived, described, conducted and presented in a very readable and comprehensive narrative. Next to the hand-full of known interactors serving as method validation, the study identifies an exhaustive list of >300 new candidate host factors of which more than 100 are shared between 2 human and 1 hamster cell line, including cells with a functional KO for G3BP1 dependent stress granule (SG) formation. Latter is importance to demonstrate SB independent nsP3 host-interactions. Top hits are functionally validated by siRNA mediated knockdown, identifying two major host protein clusters (cellular transcription/translation machinery, and membrane/cytoskeleton remodeling) essentially involved in SFV (and by extension Chickungunya virus) replication. One thus identified factors, eIF4G1 is confirmed to serve as proviral factor for SFV protein synthesis by selective inhibition of SFV replication and protein synthesis using selective small molecule inhibitor 4E1RCat.

Response: The specific responses can be found in the next section. The line numbers refer to the marked-up manuscript.

**Part II – Major Issues: Key Experiments Required for Acceptance**
Please use this section to detail the key new experiments or modifications of existing experiments that should be absolutely required to validate study conclusions.

Generally, there should be no more than 3 such required experiments or major modifications for a "Major Revision" recommendation. If more than 3 experiments are necessary to validate the study conclusions, then you are encouraged to recommend "Reject".

Reviewer #1: (No Response)

Reviewer #2: The authors treat U2OS cells with the compound CC-90009 that targets eRF3A, one of the significant antiviral hits in the siRNA screen, for degradation and do not observe an effect on SFV infection. The authors should show that CC-90009 treatment results in a decrease in eRF3A protein level by western blot and compare this to the decrease in eRF3A protein level realized by siRNA knockdown, as to explain the large difference between results from Fig. 6A and Fig. 7A.

Response: Western blotting data (with quantification) for eRF3A have been added both for the siRNA treatment and for the experiments with compound CC-90009. These are shown in the new supplementary S4 Fig. Both treatments cause a major reduction in the level of eRF3A, although the effect of siRNA is somewhat stronger. This could explain why the siRNA treatment increase virus replication while CC-90009 has no effect, but there are alternative explanations. For instance, the compound could cause wider effects, thus counteracting the proviral influence. As we have no evidence, we prefer not to speculate on the causes of the different effects (see lines 273-279 for the modifications made).

The figures should be condensed into fewer figures and data should be exchanged between the supporting information and the main manuscript to increase coherence. Fig. 1 and 2 should be combined, focusing on the setup of the proximity labelling approach. Fig. 3 is informative, but the limited conclusions drawn from the blots does not warrant its presence in the main manuscript and should be moved to the supporting information. Fig. S4 should be added to Fig. 6 to showcase the broader implication of the identified hits in alphavirus replication. This is especially important because the effect of eRF3A knockdown differs between the SFV and CHIKV. The current data presented in Fig. 7 should be condensed to two panels (see minor comments) and Fig. 8 and Fig. S5 should be added to Fig. 7, so that the mechanistic data about translation are presented collectively.

Response: We are grateful for these suggestions that will be helpful for the readers. They have been implemented as follows. Fig. 1 and Fig. 2 have been combined, as requested. However, the old Fig. 3 (current Fig. 2) we have decided to keep as a main figure (moving to supplement was suggested). In our opinion, this figure provides an immediate visual confirmation of the successful biotinylation of host proteins, and the isolation of host proteins for proteomics. It shows the physical basis for all the subsequent proteins lists and bioinformatic analyses.

Again, as suggested, the old S4 Fig has been combined with old Fig. 6 (now together comprising Fig. 5), combining the siRNA analyses for SFV and CHIKV in the same figure. In Fig. 7 (now Fig. 6) the panels have been condensed. Old Fig. 8 and Fig. S5 have been incorporated to this Fig (now Fig. 6C and D), so that all the data concerning translation are shown in the same figure.

The protein translation assay presented in Fig. S5 contains important results on the selectivity of the effect of 4EIRCat treatment on viral versus host translation. The authors should add these data to the main manuscript and perform quantitation on the lane intensities of three independent experiments to support the statement in Line 344 that host translation is restored following 4EIRCat treatment.

Response: The translation assay results have been moved to main manuscript (new Fig. 6D) as explained above. Quantitation of the translation experiments (numerical data is derived from four independent experiments) has been included.

Reviewer #3: 1. Results are discussed in a comprehensive way. The authors may however need to mention and discuss limitations of their study, including chosen methodology, issues of validation, and lack of further insight in molecular mechanisms and remaining questions at which specific steps in the viral life cycle these host factors may be involved.

Response: As suggested, we have added a paragraph to the end of the discussion section (lines 386-393) that outlines some of the limitations of the study.

2. Gene ontologies (GOs) are used to classify thus identified host factors. The authors should discuss how relevant and reliable such GOs classifications (which are defined based on their physiological role) can be considered seen that viral machinery may hijack them in a non-canonical function.

Response: This is true, and we have added a new sentence regarding this (lines 201-204). In the recent paper on alphavirus RNA binding proteins (new ref 44), Kamel et al find precisely such moonlighting roles for some nuclear proteins, including the nsP3 interactor SF3B3, which we independently discover in the current manuscript as an alphavirus host factor.

**Part III – Minor Issues: Editorial and Data Presentation Modifications**
Please use this section for editorial suggestions as well as relatively minor modifications of existing data that would enhance clarity.

Reviewer #1: Line 61: “which seems to consist of nsP3”. This statement alone is rather vague. Perhaps the authors can briefly add what observations make this likely.

Response: This is based on the recent structural data and interpretations by Kril et al. (ref. 16) as now explained on lines 59-60.

Lines 145-155: It is unclear from the text what functions are restored by the truncated G3BPs and what the replicative fitness of SFV is in G3BP1/2 knockout cells with or without expression of the truncated proteins. Please clearly provide this information either as experimental data or with citations to previous work.

Response: Better descriptions of SFV replication in the different cell lines are now provided on lines 136-139. This data is presented in the experiments of Götte et al. (Ref. 29) with these cell lines and alphavirus infection.

Consider rephrasing lines 148-150: “We used the double knock-out cell lines GFP-G3BP1 and GFP-G3BP1(1-135), reconstituting either the full-length G3BP1, or only the N-terminal NTF2-like domain, each fused with green fluorescent protein” to clarify that double knockout lines were reconstituted with G3BP1, G3BP1 (1-135) or the NTF2-like domain fused to GFP.

Response: The cell line reconstitution and nomenclature has now been more clearly explained on lines 131-136, as follows. “We used the double knock-out cell lines (∆∆G3BP1/2) that were reconstituted either with full-length G3BP1 or with the short N-terminal NTF like domain of G3BP1, termed GFP-G3BP1(1-135), each fused with green fluorescent protein. These cell lines are referred to as GFP-G3BP1 and GFP-G3BP1(1-135), respectively.”

Line 169: “Comparing the patterns between GFP-G3BP1 and GFP-G3BP1(1-135) cells”. Do you mean G3BP1/2 knockout cells that express GFP-G3BP1 and GFP-G3BP1(1-135)?

Response: Yes, this is what we mean. We hope that the modifications made in response to the previous point will make this nomenclature clear.

lines 249-250: “We selected the top high-confidence hits from the interactome and studied their effect on SFV infection in U2OS cells”. Please specify selection of this top. Is this referring to the top50 mentioned in line 190?

Response: Yes, this refers to the same top 50, also listed in S2 Table (as now better specified on line 157).

Line 327: “In contrast, 4E1RCat significantly reduced SFV replication (Fig 7B) in non-toxic concentrations.” Significance is not indicated in text, figure legend or the figure itself.

Response: Significance has now been indicated in the text at the end of this sentence.

Fig. 7A and B: There seems to be an error in the presentation of the error bars, vertical lines are missing in some of the bars.

Response: Apologies for our mistake, the error bars had gone awry when the figures were inserted to the single manuscript file initially submitted. The figures (now Fig. 6A and B) have been corrected to show the error bars.

Reviewer #2: The authors should consider adding the individual data points of the independent experiments to their graphs.

Response: In curves of inhibition and virus growth, we think that standard deviation gives a visually clearer impression. In bar graphs with few individual bars, the individual data points could be shown while maintaining clarity, but we have now consistently used standard deviation in all the figures. Individual data points for the graphs can be found in the S1 data supplement.

Responses to the points below are given immediately after each point (often simply indicated as ‘DONE’).

• Line 79: specify what is meant by ‘normal’. Response: this sentence has been modified to remove the word normal, and we now consistently refer to ‘stress granules’. The alternative structures induced by nsP3 are now consistently referred to as ‘foci’, also in the discussion section. Fros et al. (ref. 27) list four characteristics they have used to distinguish the nsP3-induced foci from stress granules.
• Line 85: add comma after specifically. DONE
• Lines 86-88: add reasoning on what the benefit is of analysing the nsP3 interactome in the absence of stress granules. Response: This allows demonstration on stress granule-independent nsP3-host interactions (line 83-84).
• Line 107: why was such a high MOI chosen to analyse viral replication, especially during replication assessment in Fig. 1C? Generally, these assessments are performed at low MOI. Response: For viruses expressing biotin ligases, the only applications (to our understanding) are in proteomics. In these experiments, high MOI permits the synchronous infection of all the cells. Low MOI would give lower signals or require longer infection or biotin labeling times (likely yielding comparatively higher background). The text has been modified on lines 115-117. High MOI represents the conditions used throughout the manuscript.
• Fig. 1.
o 1C: Y-axis label should spell titer for consistency. DONE
o Figure legend: state which MOI was used. State what the data points in 1C represent, i.e., mean ± SD of how many independent replicates. DONE
o Consider adding a schematic representation of SFV-nsP3-mTB showing the location of mTB and the different domains of nsP3. Response: We did consider this when preparing the manuscript. It may be helpful to the readers, and we have now included the scheme in S1A Figure.
• Fig. 2.
o 2A+B: write the label Streptavidin vertically and add vertical line to increase clarity. DONE (now Fig. 1 C and D)
• Line 153: repeat which time point was chosen eventually for the MS analysis in U2OS. Response: The time points have been given in Materials and Methods, and we have added a reference to that section here (now line 143).
• Fig. 3.
o 3A,B,C: add streptavidin as staining label (similar to Fig. 2). DONE (now Fig 2)
o Capitalize the P in nsP3 in the lane names. DONE
• Fig. 4.
o 4A: the shaded purple area results in the color-coded data points being difficult to differentiate. Consider removing the purple box as the dotted purple lines with labels are clear already. Be consistent in capitalization and use of spaces in figures (Fold change vs Fold Change; spaces in Fold change label but not in p-value label). DONE: the purple box has been removed in all figures of this type, and the texts are consistently presented.
o 4B+C: be consistent with labelling, choose either “not present” or “missing”. DONE
o Line 183: exchange “protein numbers” for “number of proteins”. DONE
• Line 229: add “have *been previously shown” and “*are important”. DONE
• Fig.5.
o Add FDR to axis label with (-log10 P) as unit in between brackets. DONE
o Remove grammar mistakes from figure legend. DONE
• Line 249: add that these were the top hits from the BHK-21 interactome. DONE
• Line 251: add on which location in the viral genome the luciferase is encoded. DONE
• Line 258: replace “the reduction” with “a reduction”. DONE
• Line 297: adjust to “effect on CHIKV replication”. DONE
• Fig. 6.
o Line 309: exchange average for mean. DONE
o Line 310: adjust to “error bars indicate”. DONE
o Line 314: add punctuation. DONE
• Line 322: repeat that eIF4G1 was proviral for SFV and eRF3a was antiviral for SFV to improve clarity. DONE (now line 270)
• Line 323: it is confusing to use GSPT1 and eRF3a interchangeably. Consider referring to eRF3a/GSPT1 from the start of the manuscript. Response: This sentence has been rephrased for clarity. We have used eRF3A throughout the manuscript (mentioned a dozen times), since this name better points to its role as a translation factor. Here (now line 271) the name GSPT1 is given once, in parenthesis, since it has been used in the cited paper describing the effect of the compound CC-90009 (ref. 58).
• Fig. 7.
o Apart from the major comments regarding this figure, mentioned above, the authors should condense the data presented here. It seems that the data in panel B and C are exactly the same. Panel B can be removed and an SD should be added to panel C and D. Panels C and D can be combined using left and right Y-axes. Cell viability data of CC-90009 should be added. Response: As suggested, panel B has been removed, and the new panel B is a combination of old panels C and D, in which the error bars are now shown correctly. Viability data for CC-90009 has been added as Supplementary S4C Fig, showing no effect.
• Line 356: the authors do not show data on the detection of both direct and indirect interactors and should nuance this statement. DONE
• Line 400: add “a” to proviral effect. DONE
• Line 401: it is unclear what is meant by “interactions in RNA metabolism”, please rephrase. DONE
• Line 409: similar to the minor comment about the word “normal” in the introduction, can the authors define what they mean by that. DONE (see the very first bullet point above)
• Line 415: was USP10 still present in the GFP-G3BP1(1-135) cells in general or in the interactome dataset? Please specify. DONE
• Line 419: what is meant by “USP10 could potentiate the effects of nsP3” please clarify. DONE (now lines 355-356).
• Lines 426-428: see the major comment: if CC-90009 treatment would be in accordance with the siRNA screen, replication would have significantly increased, which is not the case. Response: Rephrased.
• Line 429: “can be modified” is vague, please specify. DONE: this phrase has been removed.
• Line 437: add “complex” to end of sentence. DONE
• Line 443: can the authors expand on the use of non-classical translation initiation pathways used by host cells. Response: As individual mRNAs may have different properties (and some stress-related mRNAs are atypical in their translational properties), we think that this discussion would be lengthy and not informative in the context of our study.

• Line 444: is there an indication that compounds targeting eIF4F could be safely used in vivo without causing adverse effects? Response: This is naturally a major concern for any future utilization of these compounds. 4E1Rcat has only been studied in vitro, which we now mention here explicitly (lines 382-383). Interestingly (not added to the manuscript, as there is no published data), the compound CC-90009, which was not active against alphaviruses, but was found by others to inhibit Ebola and related viruses, has been in clinical trials as a cancer treatment, but the results were not posted, see <https://clinicaltrials.gov/study/NCT04336982> (there are two other studies in the database with results not posted), indicating that translation-modifying substances can be considered clinically interesting.
• Line 474: add space between LR2006 and OPY1. DONE
• Line 544: typo, *samples. CORRECTED
• Line 636: add space after (B). DONE
• Fig. S1: add that beta-actin was used as loading control to figure legend. DONE
• Fig. S2: see comments regarding Fig. 4A. IMPROVED as in Fig. 4A.
• Fig. S3: add that ATP levels were measured to the figure legend. DONE for S3 and the new viability data in S4C.
• Table S4: add the final concentration of the commercial antibodies. ADDED to the table.

Reviewer #3:

1. Line 14: I think “confirmed” would be more appropriate instead of “identified”. Response: edited as suggested.

2. Figures: The authors use different fonts (with and without serifs, font sizes) in graph legends (e.g. Fig.1A,B vs.Fig.4 B ,Dvs. A,C). I suggest to harmonize.

Response: Fonts have been harmonized, giving more uniformity to the figures.

3. In Fig. 4A yellow dots are hard to see.

Response: The color scheme has been altered to darker shades.

4. Fig.5 shows fold enrichment using circles which are hard to distinguish seen the small size differences. Again the yellow circle (for SG assembly) is hard to see.

Response: The color scheme has been altered for better visibility.

5. Line 270: …since siRNA silencing it induced a noticeable … Response: edited

6. Line 292: discrepancy between CHIKV and SFV restriction and proviral activity needs to be discussed. Can discrespancies e.g. in much higher fold-reduction in CHIKV replication be explained by diffenrces in replication kinetics of both viruses?

Response: This should not simply be due to the slower replication of CHIKV as compared to SFV, since the multiplicities and time points for luciferase measurements have been adjusted to obtain a roughly similar replication stage for the two viruses (Materials and Methods, section on siRNA transfection and measurement of virus replication). This sentence has been added to the manuscript (lines 256-258). It seems to be the case, as we mention in the immediately following sentence, that CHIKV is often more sensitive to reduction or absence of particular host factors. This is seen also with respect to G3BPs (as described in the Results, when the G3BP knock-out cells are introduced), as well as in other cases (refs. 20 & 29).

7. Fig. 6B. Please increase font size. Response: done.

8. EC50 and CC50 values for 4E1RCat should be rounded to maximally one digit (8.0 and 38.1µM, or 8 and 38µM) see that they are derived from curve fits and SI will not essentially change.

Response: Rounded as suggested, which is of course appropriate.

9. Fig.7: Please harmonize graphs. All graphs should have the same y-axis and should repot the same (relevant) concentration scale. Please consider reporting EC (inhibition) and CC (viability) values in combined graphs. This provides a direct visualization of specific activity (selectivity) versus unspecific.

Response: The graphs have been harmonized and the EC and CC values have been plotted in the same graph, now Fig. 6B, as suggested.

10. Fig.S5 is a nice control for specific dependency of viral protein expression on cap-dependent translation. However, densitometry of bands/smears should be provided for quantitative support. Also rest of Western blots may benefit from formal quantification.

Response: quantification has been added to this image, now main Fig. 6D. We have also quantified the reduction of viral protein nsP3 translation when the same inhibitor was used, now seen in Fig. 6C. Third, the Western blots showing reduction of cellular eRF3A levels (new Fig. S4, requested by Reviewer 2) upon different treatments show quantitative data.

11. Line 426: “In accordance” is not correct. Use of CC-9009 as chemical probe does not confirm eRF3A as host factor. Response: rephrased to “secondly”.

12. Table S4: Please provide working dilution for antibodies. Response: added to the table S4.
